# Correction to: New beginnings for dead ends: polyploidy, -SSE models and the dead-end hypothesis

**DOI:** 10.1093/aob/mcae215

**Published:** 2024-12-18

This is a **correction** to:

Eric R Hagen, Jeremy M Beaulieu, New beginnings for dead ends: polyploidy, -SSE models and the dead-end hypothesis, *Annals of Botany*, 2024;, mcae143, https://doi.org/10.1093/aob/mcae143

In the originally published version of this manuscript, Figure 2 mistakenly listed the incorrect colours in the caption. The caption should read as follows: “Solanaceae phylogeny with tip states and turnover tip rates. Tip states for ploidy are shown in green (diploids) and orange (polyploids). Tip states for breeding system are shown in blue (self-incompatible) and red (self-compatible). Turnover rates for each species are shown on the right, with colour varying from black to light yellow as rates increase.”

This error has been corrected.

